# Spontaneous adrenal pheochromocytoma rupture complicated by intraperitoneal hemorrhage and shock

**DOI:** 10.1186/1749-7922-6-27

**Published:** 2011-08-15

**Authors:** Joseph S Hanna, Philip J Spencer, Cornelia Savopoulou, Edward Kwasnik, Reza Askari

**Affiliations:** 1 Department of Trauma, Burns, Surgical Critical Care, Brigham and Women's Hospital, 75 Francis St., Boston, 02115, USA; 2 Harvard Medical School, 25 Shattuck Street, Boston, 02115, USA; 3 Brigham and Women's Surgical Associates, South Shore Hospital, 90 Libbey Parkway, South Weymouth, 02190, USA; 4 Division of Surgery, St. Elizabeth's Medical Center, 11 Nevins Street, Brighton, 02135, USA

## Abstract

MEN2A is a hereditary syndrome characterized by medullary thyroid carcinoma, hyperparathyroidism, and pheochromocytoma. Classically patients with a pheochromocytoma initially present with the triad of paroxysmal headaches, palpitations, and diaphoresis accompanied by marked hypertension. However, although reported as a rare presentation, spontaneous hemorrhage within a pheochromocytoma can present as an abdominal catastrophe. Unrecognized, this transformation can rapidly result in death. We report the only documented case of a thirty eight year old gentleman with MEN2A who presented to a community hospital with hemorrhagic shock and peritonitis secondary to an unrecognized hemorrhagic pheochromocytoma. The clinical course is notable for an inability to localize the source of hemorrhage during an initial damage control laparotomy that stabilized the patient sufficiently to allow emergent transfer to our facility, re-exploration for continued hemorrhage and abdominal compartment syndrome, and ultimately angiographic embolization of the left adrenal artery for control of the bleeding. Following recovery from his critical illness and appropriate medical management for pheochromocytoma, he returned for interval bilateral adrenal gland resection, from which his recovery was unremarkable. Our review of the literature highlights the high mortality associated with the undertaking of an operative intervention in the face of an unrecognized functional pheochromocytoma. This reinforces the need for maintaining a high index of suspicion for pheochromocytoma in similar cases. Our case also demonstrates the need for a mutimodal treatment approach that will often be required in these cases.

## Background

Multiple endocrine neoplasia 2A (MEN2A) is a rare autosomal dominant syndrome caused by missense mutations in the RET proto-oncogene associated with medullary thyroid cancer, pheochromocytoma and hyperparathyroidism. Pheochromocytoma is a rare catecholamine-secreting tumor of the adrenal glands most often presenting with the characteristic symptoms of paroxysmal hypertension, palpitations, diaphoresis, and headache. Acute onset abdominal pain and nausea may be the only presenting symptoms of spontaneous intra-abdominal hemorrhage, a rare and highly lethal complication. We present a case of spontaneous intra-abdominal hemorrhage secondary to a ruptured pheochromocytoma, subsequent management, and a review of the literature.

## Case Presentation

**Figure 1:**
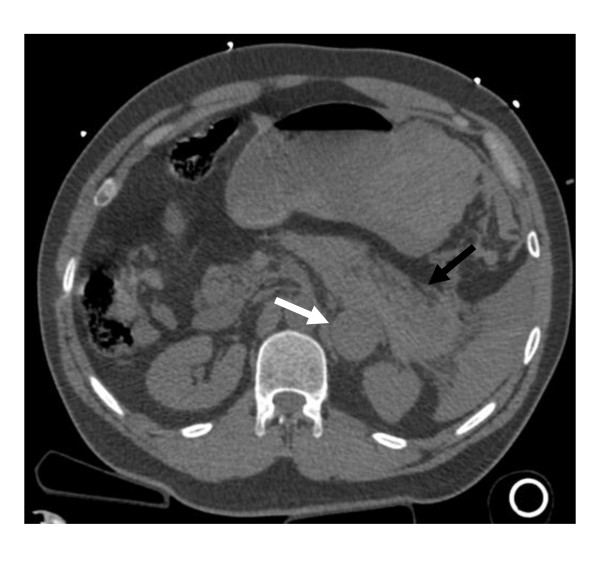
**CT scan of the abdomen with left adrenal mass (white arrow) and associated intra-peritoneal hemorrhage (black arrow) obtained on presentation to the outside hospital**.

M.J., a 38-year-old man developed sudden severe abdominal pain, nausea, and vomiting after shoveling snow. Prior to this event, he denies having had any episodes of hypertension, tachycardia or diaphoresis, although several months prior he was diagnosed with essential hypertension and was started on lisinopril. In addition, he denied any recent abdominal or flank trauma. Of note, his past medical history is significant for a diagnosis of MEN2A which was made at the age of 18 months, and a prophylactic total thyroidectomy at age 10 secondary to elevated serum calcitonin levels. Since that time he has had no further follow-up, although of his two children, his daughter has been diagnosed with MEN2A and undergone a prophylactic total thyroidectomy 2 years prior to this event. On arrival, paramedics found him near syncopal and diaphoretic with a heart rate of 180 bpm and systolic blood pressure of 64 mmHg. Fluid resuscitation was initiated and the patient was taken to an outside hospital. Initial evaluation at the local level II trauma center was notable for a heart rate of 150 bpm, systolic blood pressure of 70 mmHg, diffuse peritoneal signs, a hematocrit of 34%, INR of 1.0 and PTT of 30.4. Following resuscitation with additional crystalloid and 2 units of packed red blood cells (pRBC), his hematocrit was 34%, INR 2.4 and PTT 66.2. A non-contrast abdominal computed tomogram revealed bilateral adrenal masses and a large amount of intra and retroperitoneal hemorrhage (Figure 1). In light of his hemodynamic instability and CT findings, emergent laparotomy was undertaken. Upon entering the abdomen, a large amount of blood was encountered and immediate control of the abdominal aorta was obtained to manage the ongoing hemorrhage and facilitate resuscitation which ultimately required 12 units of pRBCs, 4 units of fresh frozen plasma (FFP) and 6 units of platelets. A bleeding source was identified in the left upper quadrant (LUQ) in the retroperitoneal fat which was oversewn. The abdomen was packed with laparotomy pads and closed; the blood loss was estimated to be 8000 cc.

The patient was subsequently transferred to our facility for further care. On arrival he was intubated and sedated with a blood pressure of 90/35 mmHg, heart rate 129 bpm, Hct 36.3%, INR 2.7 and fibrinogen 117 mg/dL. On initial examination his abdomen was tense and distended, and his extremities were cold. Ongoing hemorrhage was suspected given the coagulopathy and persistent hypotension, therefore aggressive resuscitation with blood products was resumed. An initial bladder pressure of 33 mmHg along with poor urine output, hypotension and a tense abdominal examination raised suspicion for an evolving abdominal compartment syndrome; therefore a second emergent exploration was undertaken. On entry into the abdominal cavity, the right colon was found to be frankly ischemic and persistent hemorrhage from the LUQ was again noted. As the source of bleeding could not be readily identified, an emergent splenectomy was performed, and laparotomy pads were again packed into the LUQ. Once adequate control of the bleeding was obtained with packing, attention was turned to performing a right hemicolectomy. A Bogota bag with a wound V.A.C (KCI, TX) was then fashioned for temporary abdominal closure. Following closure of the abdomen, the patient suffered cardiac arrest with pulseless electrical activity. Advanced cardiac life support measures were initiated and a perfusing rhythm was obtained shortly thereafter. Given the history of MEN2A and bilateral adrenal masses, the diagnosis of occult pheochromocytoma was entertained. The blood pressure swings were controlled with phentolamine and a sodium nitroprusside infusion with good effect. The patient was returned to the surgical intensive care unit for further management.

**Figure 2:**
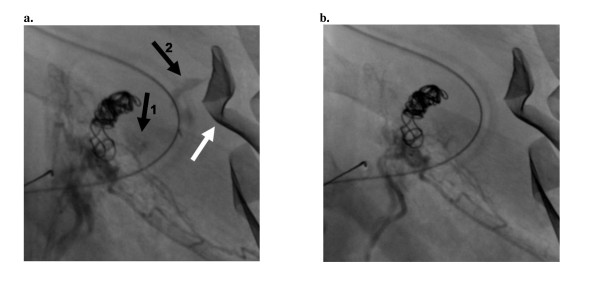
**Embolization of left adrenal artery and left T11 posterior intercostal artery**. a. Pre-embolization. The white arrow indicates a retained laparotomy pad. The coils seen left of center were previously deployed in the splenic artery stump. Black arrow #1 denotes contrast extravasation from the left adrenal artery. Black arrow #2 denotes contrast extravasation from the left posterior intercostal artery. b. Post-emboization. No further contrast extravasation was observed following embolization of both vessels with 250 micron Embozene™ (CeloNova BioSciences, GA) microspheres and Gelfoam™ (Pfizer, NY) slurry.

In the intensive care unit, the patient continued to have a labile blood pressure, a persistent base deficit, decreasing hematocrit and drainage of large amount of blood from the VAC, therefore he was emergently taken to interventional radiology. Diagnostic angiography revealed contrast extravasation from the left adrenal artery which was embolized with 250 micron Embozene™ (CeloNova BioSciences, GA) microspheres and Gelfoam™ (Pfizer, NY) slurry to good effect (Figure 2). In addition, extravasation from a left intercostal artery was also controlled with embolization. Although no active extravasation was noted from the transected end of the splenic artery, embolization was performed for additional security. Following this procedure, the patient's Hct stabilized and no further significant hemorrhage was encountered throughout the rest of his admission. Subsequently, a continuous infusion of sodium nitroprusside was required to mange the malignant hypertension. On post-operative day three, treatment with phenoxybenzamine was started for α-adrenergic blockade.

**Figure 3:**
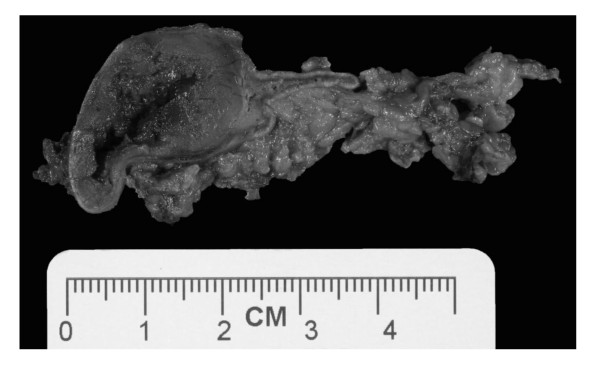
**Representative photograph of the left adrenal gland with a medullary mass and associated peri-adrenal fat**.

Serum metanephrines and normetanephrines levels were found to be markedly elevated at 14.0 nmol/L (reference range 0.00-0.49) and 24.3 nmol/L (reference range 0.0-0.89) respectively. Thereafter, his recovery was relatively unremarkable; he underwent two additional procedures to restore bowel continuity and for abdominal wall closure. He was discharged in good condition to a rehabilitation facility on hospital day 25 with instructions to continue taking phenoxybenzamine and labetolol. He returned after approximately 4.5 months for a bilateral retroperitoneoscopic adrenalectomy. Of note, intra-operatively, scarring and adhesions were noted between the left adrenal gland and surrounding periadrenal and perirenal fat. Final pathologic examination revealed a 5 cm right and 4 cm bi-lobed left adrenal (Figure 3) pheochromocytomas without evidence of definite vascular invasion or extension beyond either gland. He has since been seen in clinic for routine follow-up, and found to be recovering well, requiring labtelol 100 mg PO bid for adequate blood pressure control. He is currently taking hydrocortisone, 10 mg bid for steroid replacement.

## Discussion

Multiple endocrine neoplasia type 2A (MEN2A) or Sipple Syndrome is an autosomal dominant syndrome, first described by Sipple [1] and later characterized in multiple kindreds by Schimke [2], caused by misense mutations in the RET protooncogene [3,4], a tyrosine kinase receptor. MEN2A is characterized by the early development of medullary thyroid cancer, and later development of pheochromocytoma and primary hyperparathyroidism. The estimated prevalence of MEN2A is 2.5 per 100,000 [5] of which approximately 5-9% are sporadic and paternal in origin [6]. The incidence of pheochromocytoma in MEN2A kindreds is approximately 40-50%, with an average age of diagnosis between 30-40 years [7]. Although pheochromocytoma is traditionally referred to as the "10% tumor" (10% being bilateral, malignant, extra-adrenal, hereditary, arising in children), in MEN2A patients, approximately 68% will have bilateral involvement with malignant disease occurring in 4% of cases [8].

Pheochromocytomas are rare, catecholamine secreting, yellowish-brown tumors composed of chromaffin cells derived from embryonic neural crest cells which were first described by Frankel [9] in 1886 in a young woman likely afflicted with MEN2 [10]. Hereditary causes account for 20% of cases, while sporadic cases occur with an estimated prevalence of 0.95 per 100,000 adults per year [11]. In addition to MEN2, von Hippel Lindau Type 2, von Recklinghausen's neurofibromatosis type 1, and familial paragangliomas are associated with the development of pheochromocytomas. Eighty percent of all pheochromocytomas arise within the adrenal medulla, while extra-adrenal lesions are most commonly found in the sympathetic ganglia as well as the organs of Zuckerkandl. Of note, it is estimated that 5% of adrenal incidentalomas are likely pheochromocytomas [12]. In addition to secreting the catecholes dopamine, epinephrine and norepinephrine, numerous other hormones have been isolated from pheochromocytomas including adrenocorticotropin, vasoactive intestinal peptide, neuropeptide Y, IL-6, calcitonin, and chromogranin A.

Classically patients initially present with the triad of paroxysmal headaches, palpitations, and diaphoresis accompanied by marked hypertension. Of interest, it is estimated that pheochromocytomas are present in 0.1-0.6% of patients with hypertension [13]. In addition to these symptoms, pallor, nausea, flushing, anxiety or a sense of doom, palpitations and abdominal pain can be part of the constellation of presenting symptoms. More ominously, patients may present in fulminant cardiogenic shock [14], multiorgan failure, or with acute hemorrhage. Several biochemical assays are available to facilitate diagnosis, however, plasma free metanephrines had the highest sensitivity and urinary VMA had the highest specificity in a recent multicenter cohort trial [15] in the detection of pheochromocytomas. Once biochemical evidence of pheochromocytoma is obtained, imaging for localization should be undertaken to guide surgical resection. Computed tomography and magnetic resonance imaging provides high sensitivity for lesion detection, though poor specificity. Alternative imaging modalities such as I^123 ^or I^131 ^MIBG scintigraphy or PET may be utilized when CT or MRI fail to reveal the lesion or if malignancy is suspected.

Although both Roux (Switzerland) and Mayo (US) are credited with concomitantly performing the first successful resections of pheochromocytomas in 1926, neither described any peri-operative hemodynamic instability, and both patients survived [16]. However, in the ensuing years, further attempts at resection were met with a mortality rate of approximately 25%. It was not until 1956 when Priestley recorded a case series of 51 patients who underwent resection without any deaths. His success is attributable to the use of phentolamine and norepinephrine to manage the hemodynamic instability that is typically encountered [16]. Lessons learned during the early years of surgical management have led to the recognition of the importance of initial peri-operative α-blockade and volume expansion followed by β-blockade for management of tachycardia and hypertension in anticipation of elective surgical resection.

Implementation of these management principles in the emergent setting can often be challenging as patient presentation can be widely variable, ranging from minor retroperitoneal hemorrhage with hypertension or abdominal pain to shock and impending cardiovascular collapse. In the setting of a contained retroperitoneal hemorrhage, every effort should be made to avoid emergent or urgent surgical intervention. Not surprisingly, review of the literature reveals a mortality of ~25% associated with emergent surgical intervention for contained hemorrhage; in contrast, adequate medical preparation as described above results in a mortality rate similar to that observed for elective adrenalectomy in the absence of hemorrhage. Medical optimization should include adequate blood resuscitation, correction of any coagulopathy to limit continued hemorrhage, hemodynamic support as needed, and ultimately α-blockade followed by volume expansion and β-blockade in an in-patient setting. This simplistic algorithm must be tempered by the recognition that providing supportive care in the setting of cardiovascular collapse mediated by adrenal compression from an evolving retroperitoneal hematoma and the resulting catecholamine excess may tax even the most advanced intensive care unit. Emergent surgical intervention may be considered in cases refractory to maximal medical management as recently described by May and colleagues [17] with recognition of the attendant high morbidity and mortality.

Spontaneous hemorrhage within a pheochromocytoma resulting in capsular rupture and retroperitoneal or intra-peritoneal hemorrhage has long been recognized as a rare, but catastrophic and highly lethal event. In addition, trauma [17] and medications [18,19] have also been implicated in hemorrhagic complications. In a review of the literature, we have identified 49 documented cases between 1944 and 2010 [14,17-52] of which, including this report, 12 involved spontaneous intra-peritoneal hemorrhage [19,53-61] (Table 1). Review of these twelve cases revealed that emergent laparotomy resulted in a mortality of 29%, consistent with the mortality observed prior to the routine use of pre-operative α-adrenergic blockade [16]. More telling is the 100% mortality associated with misdiagnosis and failure to control the hemorrhage in a timely fashion. Though the case number is small, these data suggest that although undertaking an emergent exploration for this indication is fraught with danger, it offers the patient the best opportunity for survival. In the absence of adequate α-adrenergic blockade in these extreme cases, the intra-operative and post-operative care must be tailored to the clinical picture as it evolves. Thus, the anaesthesia and surgical teams must be prepared to manage sudden cardiovascular collapse, fulminant heart failure, massive pulmonary edema, and ongoing hemorrhage. Immediate availability of a perfusionist and cell-saver, an intra-aortic counter-pulsation pump, a percutaneous right ventricular assist device, a ventilator capable of maintaining high positive end-expiratory pressures with advanced ventilation modes (ex. APRV, BiLevel), an established massive transfusion protocol, and interventional radiologists are vital in the successful management of these challenging cases. If the tumor is completely removed, post-operative α-blockade is not typically necessary; however, if transcatheter arterial embolization (TAE) is used as a temporizing measure, continued α-blockade becomes essential as discussed below.

**Table 1:** Features of previously reported pheochromocytomas complicated by intra-peritoneal hemorrhage

	Pt	Symptoms	Dx Known	Intervention	Outcome
Hanna 2010	38M	Shock, abdominal pain	No	Emergent exploration	alive
Li 2009	50M	HTN, abdominal pain, palpable mass	No	Delayed exploration	alive
Chan 2003	35F	abdominal pain	No	Emergent exploration	dead
Lee 1987	31M	abdominal pain, orthostasis	No	Emergent exploration	alive
Greatorex 1984	46M	HTN, CP, palpitation, HA, emesis, tachychardia	No	Emergent exploration	alive
Wenisch 1982	62F	abdominal pain, nausea, palpable mass	No	Emergent exploration	alive
Bednarski 1981	69M	abdominal pain, dyspnea	No	None	dead
van Royen 1978	53M	HTN, abdominal pain, palpable mass, bronchospasm	No	None	dead
Van Way 1976	76F	HTN, abdominal pain	Yes	Emergent exploration	alive
Gielchinsky 1972	36M	abdominal pain, peritonitis	Yes	Delayed exploration	alive
Cahill 1944	53F	abdominal pain	No	Emergent exploration	dead
	61	shock, sudden death	No	None	dead

A summary of the 11 previously described cases of ruptured pheochromocytoma with free intraperitoneal hemorrhage including the present case. The relevant symptoms on presentation, timing of operative intervention and outcome are summarized.

In the present case, we were faced with a unique set of circumstances which dictated an unconventional course of management. Although the patient's medical history notable for total thyroidectomy as a child and the presence of the bilateral adrenal masses raised suspicion for MEN2A and possible pheochromocytoma, given his initial presentation in extremis with hemoperitoneum the decision to undertake an emergent exploratory laparotomy was warranted. Although only partial control of the hemorrhage was obtained at that time, it should be stressed that inaction may have resulted in this patient's demise given the historically observed 100% mortality in the face of conservatively managed intra-peritoneal hemorrhage from pheochromocytoma. Upon arrival to our facility, we were faced with an evolving abdominal compartment syndrome in addition to acute hemorrhage of unclear etiology. In the course of the second laparotomy, hemodynamic instability, the need to address the sequelae of abdominal hypertension, and worsening coagulopathy precluded further exploration of the LUQ for the continued source of hemorrhage. Moreover, given the presence of bilateral adrenal masses in the setting of a history of MEN2A, further exploration of the adrenals without proper α-blockade presented addition significant risk of morbidity and mortality. Therefore the decision was made to proceed with angiographic embolization in the setting of continued bleeding.

TAE as a therapeutic option for pheochromocytoma was first described in 1978 by Bunuan [62] and collegues. Their effort to use gel foam TAE was met with significant hemodynamic instability resulting in emergent laparotomy for excision of the necrotic tumor. Since this initial experience, TAE has been reported in the literature as a palliative option in the management of malignant pheochromocytoma when surgical extirpation is not feasible [63,64]. More germane to the present case, the use of TAE for management of acute spontaneous intraperitoneal hemorrhage from a pheochromocytoma has not been previously reported, although its use in retroperitoneal hemorrhage as been described by two separate groups [17,50]. In the present case any further effort to explore the LUQ for the source of hemorrhage may very well have resulted in the patient's demise. We therefore elected to salvage the situation by employing damage control techniques to quickly get the patient out of the operating room to facilitate TAE of the suspected hemorrhaging pheochromocytoma. Interestingly, in addition to embolization of a left adrenal artery in this case, a bleeding left intercostal artery was also identified. In an effort to better define the anatomy of the suprarenal arteries, Toni and colleagues reviewed aortography performed on patients without known suprarenal disease [65]. They identified the origin of the left suprarenal artery as a left intercostal branch in 3% of the patients in their study. As described in all of these reports, post-TAE hypertension can present a formidable challenge. In this case, malignant hypertension was successfully managed with infusion of sodium nitroprusside in the acute setting, followed by administration of phenoxybenzamine.

## Conclusion

Spontaneous intraperitoneal hemorrhage remains a rare complication of pheochromocytoma, though the physiologic consequences present considerable medical and surgical challenges. Unfortunately, the resulting emergent clinical scenario does not allow for optimal pre-operative medical preparation with α-adrenergic blockade, and as such is associated with a high mortality. The present case has demonstrated the importance of multi-modal therapy including the need for emergent surgical intervention and the availability of interventional radiology for control of the hemorrhage. Most importantly, a high index of suspicion must be maintained in similar cases so that the highly lethal hemodynamic sequelae may be anticipated and managed with the appropriate pharmacologic agents to ensure optimal outcomes.

## Consent

Written informed consent was obtained from the patient for publication of this Case report and any accompanying images. A copy of the written consent is available for review by the Editor-in-Chief of this journal.

## List of Abbreviations

MEN2A: Multiple Endocrine Neoplasia Type 2A; pRBC: packed red blood cells; FFP: fresh frozen plasma; LUQ: left upper quadrant; TAE: transcatheter arterial embolization; APRV: Airway Pressure Release Ventilation

## Competing interests

The authors declare that they have no competing interests.

## Authors' contributions

JH participated in the surgical and critical care of this patient and drafted the manuscript. PS participated in drafting the manuscript. CS, EK and RA participated in the surgical care of this patient and critical review of the manuscript. All authors have read and approved the final manuscript.
